# Supplementary material for: Evolving epigenomics of immune cells at single-nucleus resolution in children en route to type 1 diabetes
Source: Nat Commun. 2026 Feb 25;17:3168. doi: 10.1038/s41467-026-69923-x (PMC13046956; doi:10.1038/s41467-026-69923-x)
Supplement: Supplementary file 1 — Supplementary information [file 41467_2026_69923_MOESM1_ESM.pdf]

SUPPLEMENTARY INFORMATION

Evolving epigenomics of immune cells at single-nucleus resolution children en route to type 1 diabetes

Tomi Pastinen, Elin Grundberg, Todd Bradley, Jarno Honkanen, Warren Cheung, Arja Vuorela, Jeff J. Johnston, Byunggil Yoo, Santosh Khanal, Rebecca McLennan, Jorma Ilonen, Outi Vaarala, Jeffrey P. Krischer, and Mikael Knip

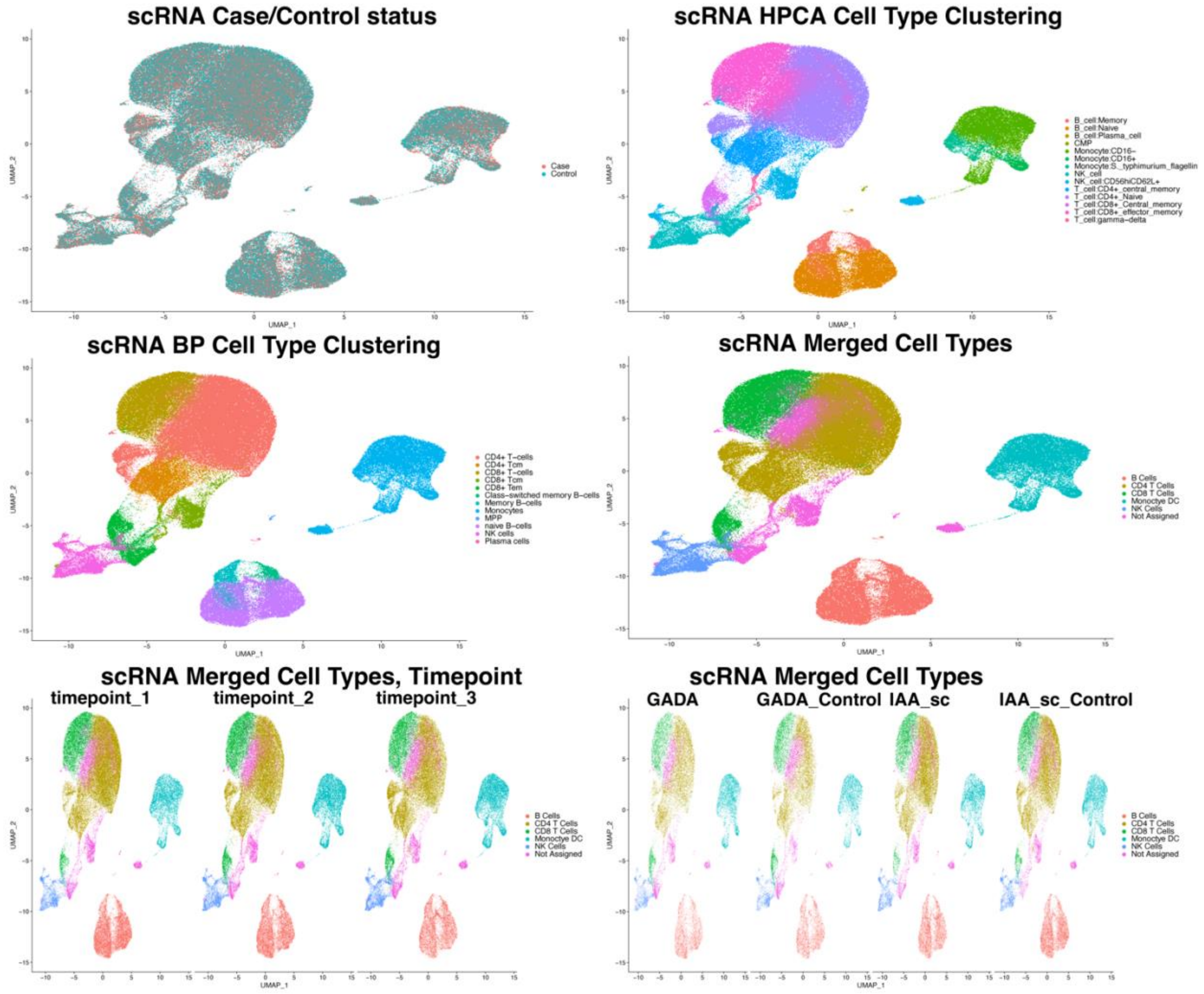

Supplementary Figure 1: **Labelled single cell UMAP visualizations for scRNA**. (**top left**) UMAP of scRNA cases (orange) and controls (teal). (**top right**) UMAP of scRNA HPCA cell type clustering. (**middle left**) UMAP of scRNA BP cell type clustering. (**middle right**) UMAP of scRNA merged cell types. (**bottom left**) UMAPs of scRNA merged cell types at the 3 timepoints. (**bottom right**) UMAPs of scRNA merged cell types of diabetes-associated autoantibodies and controls. Link to code to generate UMAPs is provided.

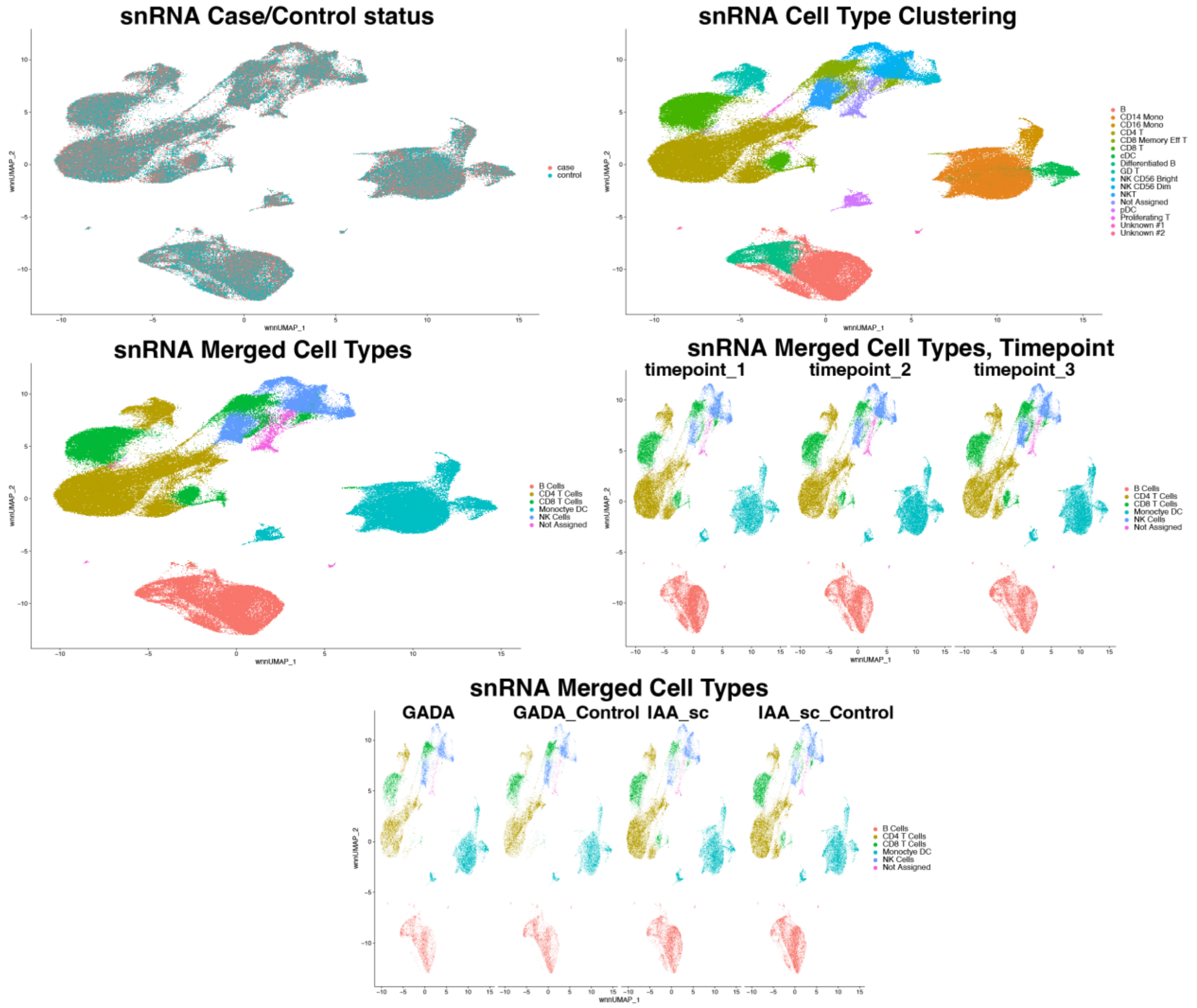

Supplementary Figure 2: Labelled single cell UMAP visualizations for snRNA. (**top left**) UMAP of snRNA cases (orange) and controls (teal). (**top right**) UMAP of snRNA cell type clustering. (**middle left**) UMAP of snRNA merged cell types. (**middle right**) UMAPs of snRNA merged cell types at the 3 timepoints. (**bottom**) UMAPs of snRNA merged cell types of diabetes-associated autoantibodies and controls. Link to code to generate UMAPs is provided.

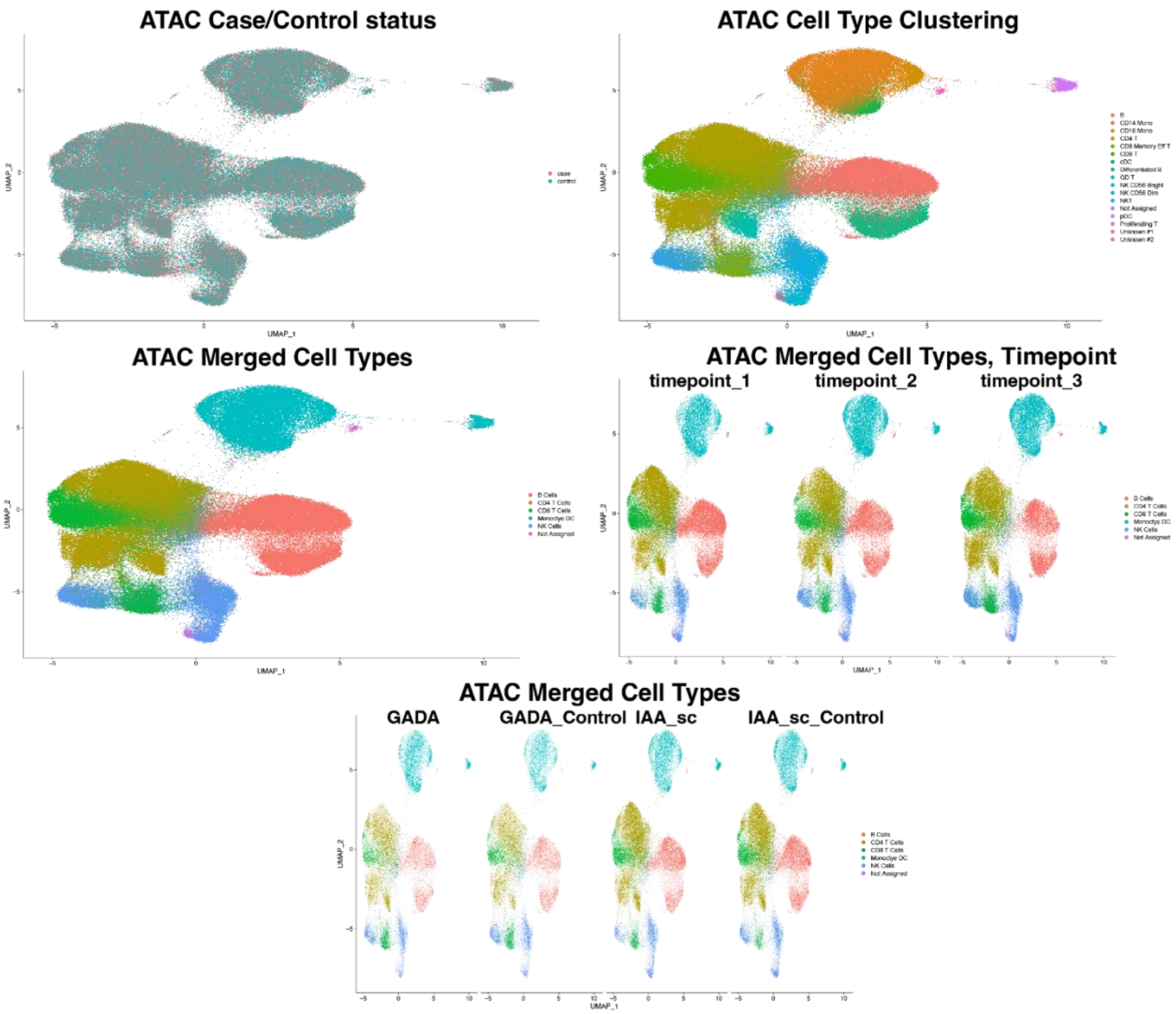

Supplementary Figure 3: Labelled single cell UMAP visualizations for ATAC. (**top left**) UMAP of ATAC cases (orange) and controls (teal). (**top right**) UMAP of ATAC cell type clustering. (**middle left**) UMAP of ATAC merged cell types. (**middle right**) UMAPs of ATAC merged cell types at the 3 timepoints. (**bottom**) UMAPs of ATAC merged cell types of diabetes-associated autoantibodies and controls. Link to code to generate UMAPs is provided.

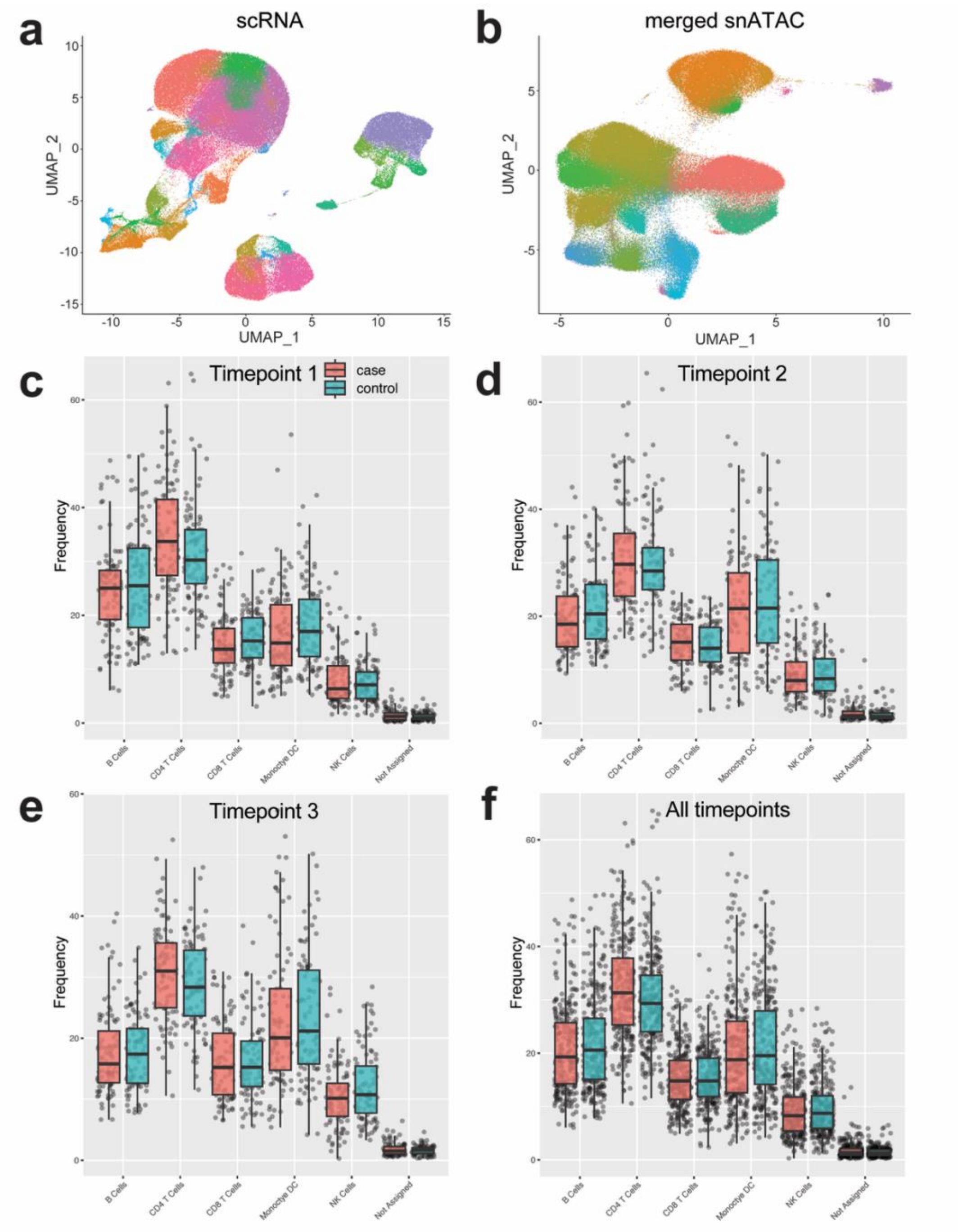

Supplementary Figure 4: Major cell and nuclei populations observed in cases and controls. Code to generate UMAPs and Source Data are provided in the Source Data file. **a)** Seurat analyses and clustering of independently analyzed PBMCs by scRNA sequencing. **b)** Signac analyses and clustering of merged snATAC object derived from 263,308 QC'd singleton nuclei included in separate 10X ATAC-seq. capture and 10X multiome capture. **c-f)** Proportions of major cell types inferred from cell and nuclei labeling by Seurat across all sc and sn captures in the study at all three timepoints and combined. No statistically significant differences in major cell type abundancies were observed between cases and controls. Boxplots show median, with hinges at first and third quartiles and whiskers to the largest value no further than 1.5 * IQR from the hinge.

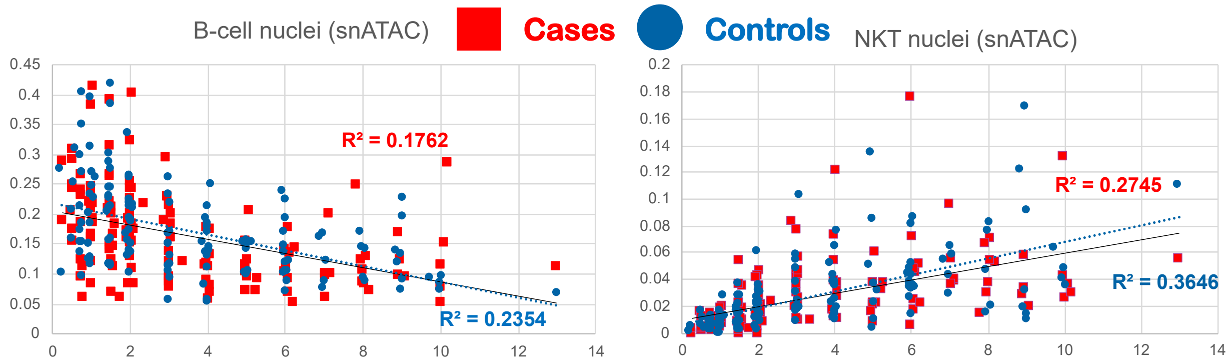

Supplementary Figure 5: Age-dependent variation in nuclei abundance. Many cell types demonstrate age-dependent abundancies. Examples (y-axis = cell-type fraction, x-axis = calendar age in years) shown for B-cell nuclei showing age dependent decline and NKT-nuclei showing age-dependent increase in abundancy. We note cases and controls are age-matched. Source Data described in the Source Data file.

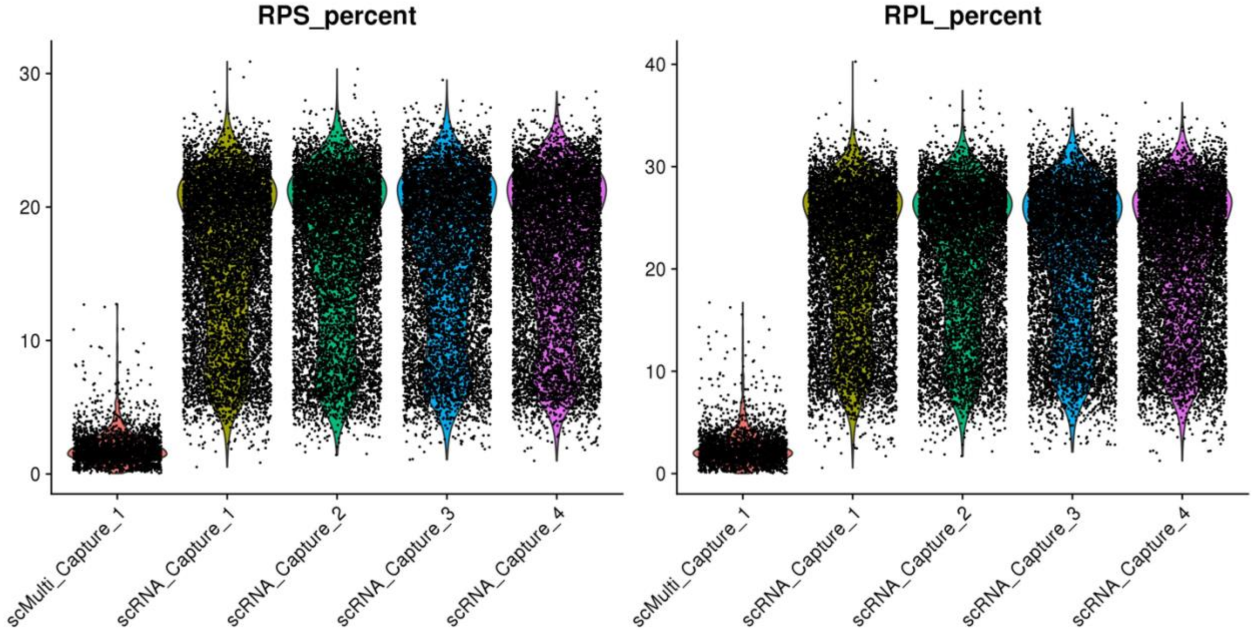

Supplementary Figure 6: Predominant depletion of ribosomal RNA species in nuclear RNA assessed by scMultiome. Four scRNA captures (scRNA_Capture1-4, x-axis) each show stable fractions of ribosomal RNA transcripts of ~50% of reads (RPS + RPL %, y-axis), whereas only 5-10% RNA reads from single nuclei from 10X multiome experiment (scMultiome_Capture1) maps to ribosomal RNAs. Source Data generated from Seurat and described in the Source Data file.

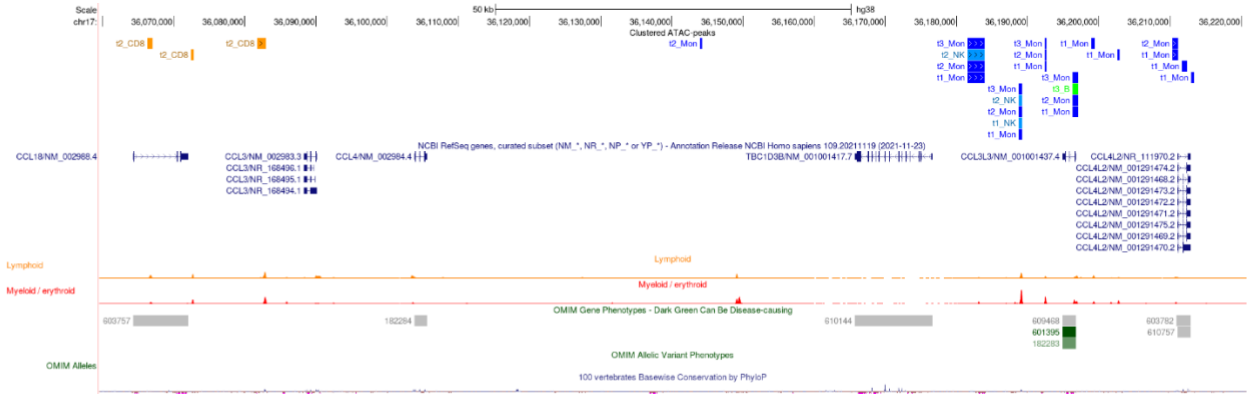

Supplementary Figure 7: Non-random genomic clustering of case versus control differential chromatin peaks. We looked for regional clustering of case vs. control differential peaks in snATAC-seq. Specifically, a total of ~1,500,000 case-control chromatin association tests (99,799 peaks x 5 cell types x 3 timepoints) were tested with 5,487 associations at qv<0.1 Observing any two signals from same 100kb windows has an empirical probability of 0.00014. We observed over 1,700 "regional peak enrichments" representing greater than 8x enrichment from random distribution. An example region in UCSC Browser snapshot shows multiple peak associations also included in Figure 2 illustrating chemokine cluster in chr17 with abundance of clustered differentially open monocyte (blue) peaks across timepoints (top track). Some regional clustering of CD8 differential chromatin (orange) is evident proximal to monocyte chromatin cluster. These differentially regulated chromatin regions overlap previously mapped DHS peaks in blood leukocytes ("Lymphoid"/Orange and "Myeloid/Erythroid"/Red tracks). Source Data described in the Source Data file.

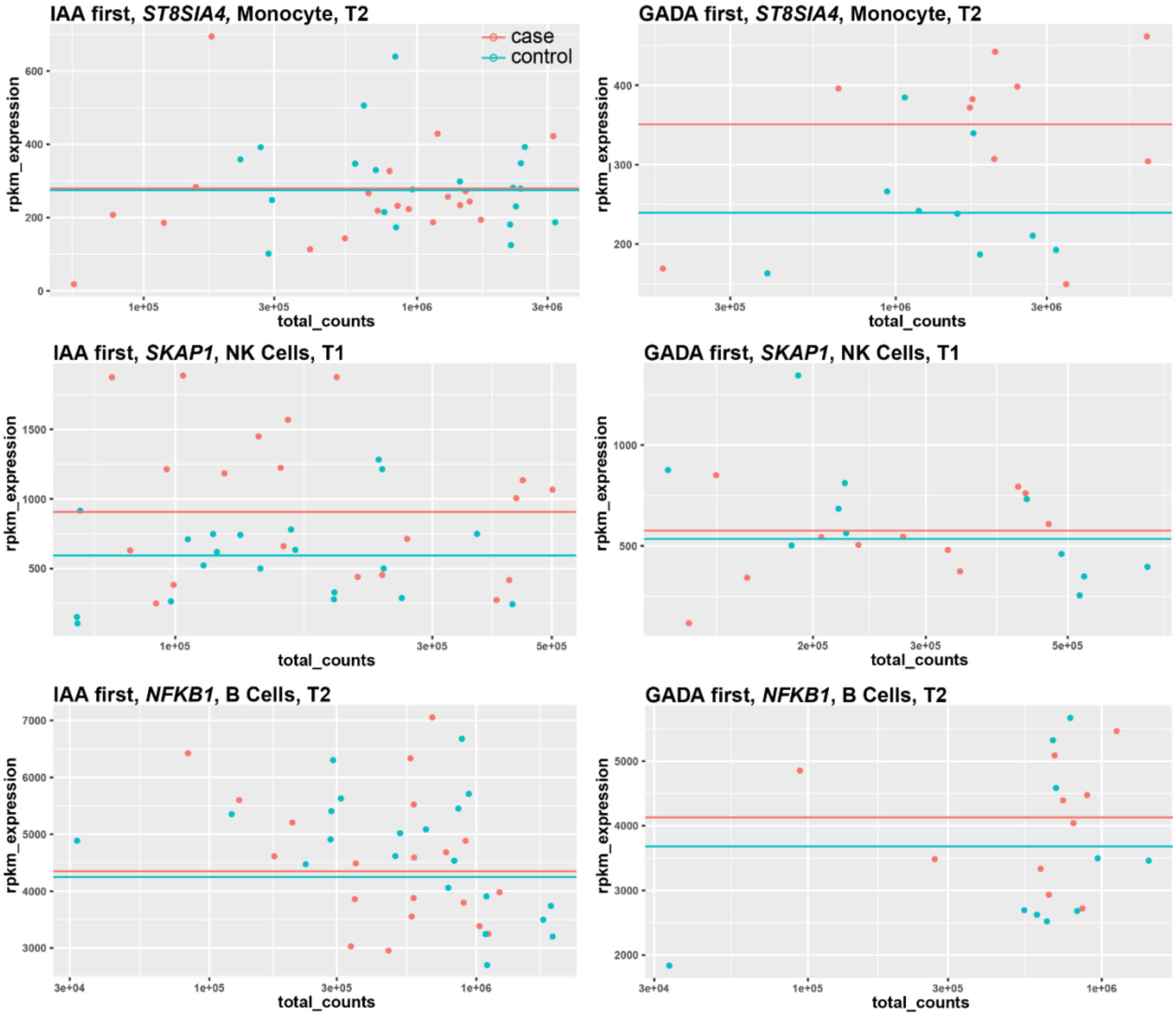

Supplementary Figure 8: Normalized expression scores and read counts across endophenotype selective case versus control differences in snRNA expression signals. Six examples of normalized (Reads Per Kilobase per Million mapped reads, rpkm; y-axis) per gene and total reads per individual (x-axis) used in statistical association to determine case (red dots) - control (green dots) differences in expression among IAA first and GADA first subpopulations. Top graphs show little or no difference in case - control expression of ST8SIA4 in monocytes at time point 2 among IAA first endotype, whereas (second from top) same gene is significantly (P=2.2e-50) upregulated in GADA first cases as compared to matched controls. Middle two graphs show a reverse situation in SKAP1 in NK-cells at time point 1 where IAA first endotype samples are significantly higher (P=1.4e-60) among cases as compared to matched controls and GADA first shows little difference among cases versus matched controls. Finally, the bottom example shows large difference (P=3.0e-30) among GADA First group in NFKB1 expression for B-cells (timepoint 2), where IAA first group is on average much closer to matched control average. Source Data described in the Source Data file.
