# Supplementary material for: Evolving epigenomics of immune cells at single-nucleus resolution in children en route to type 1 diabetes
Source: Nat Commun. 2026 Feb 25;17:3168. doi: 10.1038/s41467-026-69923-x (PMC13046956; doi:10.1038/s41467-026-69923-x)
Supplement: Supplementary file 2 — Reporting Summary [file 41467_2026_69923_MOESM2_ESM.pdf]

Corresponding author(s): Tomi Pastinen

Last updated by author(s): Jan 5, 2026

Reporting Summary

Nature Portfolio wishes to improve the reproducibility of the work that we publish. This form provides structure for consistency and transparency in reporting. For further information on Nature Portfolio policies, see our Editorial Policies and the Editorial Policy Checklist.

Statistics

For all statistical analyses, confirm that the following items are present in the figure legend, table legend, main text, or Methods section.

n/a	Confirmed	
☐	☒	The exact sample size (*n*) for each experimental group/condition, given as a discrete number and unit of measurement
☐	☒	A statement on whether measurements were taken from distinct samples or whether the same sample was measured repeatedly
☐	☒	The statistical test(s) used AND whether they are one- or two-sided<br>*Only common tests should be described solely by name; describe more complex techniques in the Methods section.*
☐	☒	A description of all covariates tested
☐	☒	A description of any assumptions or corrections, such as tests of normality and adjustment for multiple comparisons
☐	☒	A full description of the statistical parameters including central tendency (e.g. means) or other basic estimates (e.g. regression coefficient) AND variation (e.g. standard deviation) or associated estimates of uncertainty (e.g. confidence intervals)
☐	☒	For null hypothesis testing, the test statistic (e.g. *F*, *t*, *r*) with confidence intervals, effect sizes, degrees of freedom and *P* value noted<br>*Give P values as exact values whenever suitable.*
☒	☐	For Bayesian analysis, information on the choice of priors and Markov chain Monte Carlo settings
☒	☐	For hierarchical and complex designs, identification of the appropriate level for tests and full reporting of outcomes
☐	☒	Estimates of effect sizes (e.g. Cohen's *d*, Pearson's *r*), indicating how they were calculated

Our web collection on statistics for biologists contains articles on many of the points above.

Software and code

Policy information about availability of computer code

Data collection	This study included PBMCs from 98 European Trial to Reduce IDDM in the Genetically at Risk (TRIGR) participants. Cell Isolations and genomics were performed by our group as described in the methods.
Data analysis	Analysis of generated data was performed using demuxlet v2, Illumina Real Time Analysis (RTA) software and bcl2Fastq2-20, cellranger-4.0.0 and cellranger-atac-1.2.0, R-based Seurat 4.0 package, Signac (https://CRAN.R-project.org/package=Signac) workflow.

For manuscripts utilizing custom algorithms or software that are central to the research but not yet described in published literature, software must be made available to editors and reviewers. We strongly encourage code deposition in a community repository (e.g. GitHub). See the Nature Portfolio guidelines for submitting code & software for further information.

Data

Policy information about availability of data

All manuscripts must include a data availability statement. This statement should provide the following information, where applicable:

- Accession codes, unique identifiers, or web links for publicly available datasets
- A description of any restrictions on data availability
- For clinical datasets or third party data, please ensure that the statement adheres to our policy

Data are stored in the European Genome-phenome Archive (EGA, https://ega-archive.org).
https://ega-archive.org/datasets/EGAD50000001257. Individual participant data are shared in a de-identified format to protect the identity of the participants. There are no restrictions on who the data can be made available to or for which purpose. Please contact Marja Salonen (marja.salonen@helsinki.fi) to request

access. One to six working days are the expected time frame for response to access requests. The data will be available for 12 months once access has been granted. All data are included in the Supplementary Information or available from the authors, as are unique reagents used in this Article. The raw numbers for charts and graphs are available in the Source Data file whenever possible.

Research involving human participants, their data, or biological material

Policy information about studies with human participants or human data. See also policy information about sex, gender (identity/presentation), and sexual orientation and race, ethnicity and racism.

Reporting on sex and gender	Equal numbers of males and females for cases and controls were included, as described in Table 1. Sex was obtained from self-reporting at enrollment. No sex-specific analysis was performed.
Reporting on race, ethnicity, or other socially relevant groupings	Analysis specific to race, ethnicity and other socially relevant groupings were not performed.
Population characteristics	Forty-nine autoantibody-negative control subjects were matched with the forty-nine subjects who progressed to clinical T1D during the follow-up (cases) for date of birth (± 1 year) and geographical region. All TRIGR participants had a family member affected by T1D and carried an HLA genotype conferring increased risk for T1D The TRIGR participants were recruited in 2002-2007 before or immediately after birth followed by randomization to two weaning groups. The participants attended the study centers at the age of 3, 6, 9,12, 18 and 24 months and subsequently annually until the youngest child turned 10. A sample for the isolation of peripheral blood mononuclear cells (PBMC) was collected at each visit and the sample from European participants was shipped overnight for isolation in the TRIGR Core Laboratory in Helsinki. Seroconversion to autoantibody positivity was observed in the 49 cases at the mean age of 2.4 years (range 0.7 to 7.1 years) and T1D was diagnosed at the mean age of 6.6 years (range 1.0 to 13.7 years). The controls remained autoantibody negative throughout the follow-up. Each participant in the current study contributed three samples; The first time point in the cases was before seroconversion at the mean age of 1.6 years (range 0.3 to 6.1 years), the second time point soon after the seroconversion at the mean age of 3.0 years (range 0.8 to 8.2 years) and the third time point close to the time of T1D diagnosis at the mean age 6.0 years (range 1.5 to 13.0 years). The time points in the controls corresponded to similar ages in the cases.
Recruitment	The TRIGR participants were recruited in 2002-2007 before or immediately after birth at 78 study centers in 15 countries. https://www.trigr.org/centres.html . Written informed consent was obtained from the family before enrollment.
Ethics oversight	The TRIGR study was approved by the Ethical Committees of all participating centers. All uses of human material have been approved by the Ethics Committee of the Hospital District of Helsinki and Uusimaa (Helsinki, Finland, HUS 617/E0/02).

Note that full information on the approval of the study protocol must also be provided in the manuscript.

Field-specific reporting

Please select the one below that is the best fit for your research. If you are not sure, read the appropriate sections before making your selection.

[x] Life sciences [ ] Behavioural & social sciences [ ] Ecological, evolutionary & environmental sciences

For a reference copy of the document with all sections, see nature.com/documents/nr-reporting-summary-flat.pdf

Life sciences study design

All studies must disclose on these points even when the disclosure is negative.

Sample size	98 individuals were included in this study. 49 progressing to T1D (cases) and age-matched 49 non-progressors (controls) across 3 timepoints. Sample size represents that maximum number of cases we were able to obtain with longitudinal data and samples, with sufficient PBMCs available and controls to match.
Data exclusions	No data was excluded in the analysis.
Replication	Multiple orthogonal layers of data were produced from each subject, analyses presented include only datapoints that were replicated at least on one independent datalayer.
Randomization	Cases and controls were captured in large batches and each capture included the case and its' matched control to ensure that batch effects in single cell analyses were not source of case - control difference.
Blinding	Blinding was not applicable to the studies as identification of T1D status was necessary for grouping.

Reporting for specific materials, systems and methods

We require information from authors about some types of materials, experimental systems and methods used in many studies. Here, indicate whether each material, system or method listed is relevant to your study. If you are not sure if a list item applies to your research, read the appropriate section before selecting a response.

Materials & experimental systems

n/a	Involved in the study
☒	☐ Antibodies
☒	☐ Eukaryotic cell lines
☒	☐ Palaeontology and archaeology
☒	☐ Animals and other organisms
☐	☒ Clinical data
☒	☐ Dual use research of concern
☒	☐ Plants

Methods

n/a	Involved in the study
☒	☐ ChIP-seq
☒	☐ Flow cytometry
☒	☐ MRI-based neuroimaging

Clinical data

Policy information about clinical studies

All manuscripts should comply with the ICMJE guidelines for publication of clinical research and a completed CONSORT checklist must be included with all submissions.

Clinical trial registration	NCT00179777
Study protocol	https://clinicaltrials.gov/study/NCT00179777#study-plan
Data collection	The TRIGR participants were recruited in 2002-2007 before or immediately after birth at 78 study centers in 15 countries. https://www.trigr.org/centres.html Written informed consent was obtained from the family before enrollment.
Outcomes	Primary outcome of the TRIGR study was progression to T1D. Study design for the TRIGR cohort is described in reference 17

Plants

Seed stocks	*Report on the source of all seed stocks or other plant material used. If applicable, state the seed stock centre and catalogue number. If plant specimens were collected from the field, describe the collection location, date and sampling procedures.*
Novel plant genotypes	*Describe the methods by which all novel plant genotypes were produced. This includes those generated by transgenic approaches, gene editing, chemical/radiation-based mutagenesis and hybridization. For transgenic lines, describe the transformation method, the number of independent lines analyzed and the generation upon which experiments were performed. For gene-edited lines, describe the editor used, the endogenous sequence targeted for editing, the targeting guide RNA sequence (if applicable) and how the editor was applied.*
Authentication	*Describe any authentication procedures for each seed stock used or novel genotype generated. Describe any experiments used to assess the effect of a mutation and, where applicable, how potential secondary effects (e.g. second site T-DNA insertions, mosiacism, off-target gene editing) were examined.*
