# Supplementary material for: Evolving epigenomics of immune cells at single-nucleus resolution in children en route to type 1 diabetes
Source: Nat Commun. 2026 Feb 25;17:3168. doi: 10.1038/s41467-026-69923-x (PMC13046956; doi:10.1038/s41467-026-69923-x)
Supplement: Supplementary file 4 — Description of Additional Supplementary Files [file 41467_2026_69923_MOESM4_ESM.pdf]

Description of Additional Supplementary Files

File name: Supplementary Data 1

Description: Number of cells assayed from each sample (with subtype and condition), for each experiment (dataset and timepoint).

File name: Supplementary Data 2

Description: Summary grouping for each experiment (dataset and timepoint) and subtype, the number of samples and total number of cells.

File name: Supplementary Data 3

Description: Pearson correlation of scRNA gene expression vs snATAC open chromatin, across available celltypes.

File name: Supplementary Data 4

Description: Case vs Control fisher testing p-values for scRNA genes.

File name: Supplementary Data 5

Description: Case vs Control fisher testing p-values for multiomeRNA genes.

File name: Supplementary Data 6

Description: Case vs Control fisher testing p-values for ATAC peaks.

File name: Supplementary Data 7

Description: GADA vs Control fisher testing p-values for scRNA genes.

File name: Supplementary Data 8

Description: GADA vs Control fisher testing p-values for multiome RNA genes.

File name: Supplementary Data 9

Description: GADA vs Control fisher testing p-values for ATAC peaks.

File name: Supplementary Data 10

Description: IAAsc vs Control fisher testing p-values for scRNA genes.

File name: Supplementary Data 11

Description: IAAsc vs Control fisher testing p-values for multiome RNA genes.

File name: Supplementary Data 12

Description: IAAsc vs Control fisher testing p-values for ATAC peaks.

File name: Supplementary Data 13

Description: Linear model fit of multiome snRNA gene feature_tpm vs calendar age of the sample.

File name: Supplementary Data 14

Description: Replication of known T1D risk loci SNV (associations within 1Mb).

File name: Supplementary Data 15

Description: snRNA expression differences in genes with T1D-associated candidate genes.

File name: Supplementary Data 16

Description: snATAC peaks within 100kb of T1D-associated SNV

.
